# The Impact of Thoracic Trauma on Morbidity and Outcomes: A Six-Year Experience From a Tertiary Care Level 1 Center

**DOI:** 10.7759/cureus.73580

**Published:** 2024-11-13

**Authors:** Amit K Singh, Ganpat Prasad, Prabhaker Mishra

**Affiliations:** 1 Trauma, Sanjay Gandhi Post Graduate Institute of Medical Sciences, Lucknow, IND; 2 Anesthesiology, Sanjay Gandhi Post Graduate Institute of Medical Sciences, Lucknow, IND; 3 Biostatistics and Health Informatics, Sanjay Gandhi Post Graduate Institute of Medical Sciences, Lucknow, IND

**Keywords:** morbidity, mortality, pneumothorax, thorax, trauma

## Abstract

Objective: The study aimed to summarize the impact of thoracic injury on mortality and morbidity in thoracic trauma patients.

Material and methods: This is a retrospective observational study of all patients with thoracic injuries admitted between July 2018 and June 2024. The demographic profile, mechanism of injury, type of injury, injury severity score, duration of injury, hemodynamic status, surgical findings, concomitant injuries, hospitalization, intensive care unit stay, need for mechanical ventilation, and blood transfusions were recorded.

Results: A total of 1,576 patients were admitted with thoracic injuries, representing 27.4% of all trauma admissions. The most common mechanism of injury was blunt chest trauma, which was found in 1,377 patients (87.4%). Of these 1,576 patients, 1,308 (83%) were hemodynamically stable, while 268 (17%) were unstable. The majority of patients were not treated surgically. The overall mortality was 119 (7.5%). Age, number of rib fractures, time interval to admission, and need for mechanical ventilation were found to be significantly related to mortality.

Conclusion: The mortality rate for thoracic injuries depends on several factors, including age, late-onset, bilateral thoracic injuries, concomitant extrathoracic injuries, need for mechanical ventilation, and adequate pain management.

## Introduction

Thoracic trauma ranks as the second most prevalent unintentional traumatic injury that is a primary contributor to mortality globally, accounting for up to about 35% of trauma-associated mortalities [1]. It can range from rib fractures to life-threatening injuries to the lungs, sternum, heart, and vascular structures of the mediastinum. Blunt chest trauma occurs more frequently than penetrating trauma, constituting 15% of all trauma cases [2]. Thoracic injuries can progress rapidly and, therefore, require a systematic approach prioritizing airway, breathing, circulation, multimodal analgesia, and pulmonary toilet. In addition, delays in intervention and late diagnosis are associated with high mortality [3-6]. Less than 10% of blunt thoracic injuries and 15% of penetrating injuries demand surgical intervention, while the remainder could be treated by simple procedures performed by emergency medical personnel [7].

In developing countries like India, thoracic injuries are continuously increasing, and many patients die despite hospitalization [8]. The cause of increased morbidity and mortality is primarily due to delayed pulmonary complications [9]. There are many factors that influence morbidity and mortality in thoracic injuries. Along with age, these include preexisting chronic lung disease, the number of rib fractures, the presence of rib fractures, the need for mechanical ventilation, coexisting head injuries, and other extrathoracic injuries. The research objective of this study was to evaluate the prevalence, severity, risk factors, morbidity, and outcome among thoracic trauma patients admitted to a level 1 trauma center.

## Materials and methods

A retrospective analysis of data from the 210-bed level 1 Apex Trauma Centre Emergency Department at Sanjay Gandhi Postgraduate Institute of Medical Science, Lucknow, India, was conducted to evaluate the impact and outcomes in consecutive patients with thoracic injuries hospitalized from July 2018 to June 2024 after obtaining ethics approval from the Institute's Ethics Committee. While doing the study, we adhered to Strengthening the Reporting of Observational Studies in Epidemiology guidelines. Patients who had presented with cardiac arrest or reported death had been excluded from the research. As no autopsy facility was available at our institute, the records of the autopsy reports were neither traced nor analyzed. A comprehensive assessment of all patient files was performed, analyzing the results of primary and secondary surveys, intensive care unit (ICU) data, biochemical parameters, surgery records, emergency department measures, radiological investigations, and hospitalization details. Each patient has been followed up for 30 days to investigate morbidity and mortality. All patients have been managed as per the advanced trauma life support protocol. Primary and secondary surveys were performed, and high-resolution computed tomography (CT) with 3-D reconstruction was obtained for a detailed assessment of thoracic injuries. Hemodynamically unstable patients had been transferred directly to the operating theater for emergency thoracotomy, while hemodynamically stable patients had been treated conservatively. Blunt trauma is defined as injuries in which organs and structures are injured without destroying the integrity of the tissue. A flail chest is characterized by the fracture of three or more adjacent ribs in at least two locations.

Mortality is defined as death occurring during hospitalization, either from the trauma incident or from complications emerging thereafter. The duration of ICU stay is described as the interval from admission to the ICU until transfer from the ICU. The hospital stay is described as the duration from admission to discharge or death within the hospital. Ventilation hours are defined as the duration the patient has been subjected to mechanical ventilation. Hemothorax is defined as fluid between the visceral and parietal pleura that is 50% or more of the hematocrit of the peripheral blood or having 35-70 Hounsfield unit on CT chest. Retained hemothorax is defined as residual blood clots of 500 mL remaining in the pleural cavity after 72 hours of chest drainage. The Easter scoring system is used to assess morbidity, while the pain, inspiration, and cough reflex score is used to guide nonsurgical treatment (Table 1).

**Table 1:** Pain, inspiration, and cough score ^a^Goal volume is 80% of inspiratory capacity ^b^Alert volume is 15 mL/kg of ideal body weight PIC: pain, inspiration, and cough

Pain (PIC score allotted): patient-reported (pain intensity scale 0-10)	Inspiration (PIC score allotted): inspiratory spirometer; goal and alert levels set by respiratory therapist	Cough (PIC score allotted) assessed by bedside nurse
Controlled (3) (if pain intensity scale is 0-4)	Above goal volume^a ^(4)	Strong (3)
	Goal to alert volume^b ^(3)	
Moderate (2) (if pain intensity scale is 5-7)	Below alert volume (2)	Weak (2)
Severe (1) (if pain intensity scale is 8-10)	Unable to perform incentive spirometry (1)	Absent (1)

**Figure 1:**
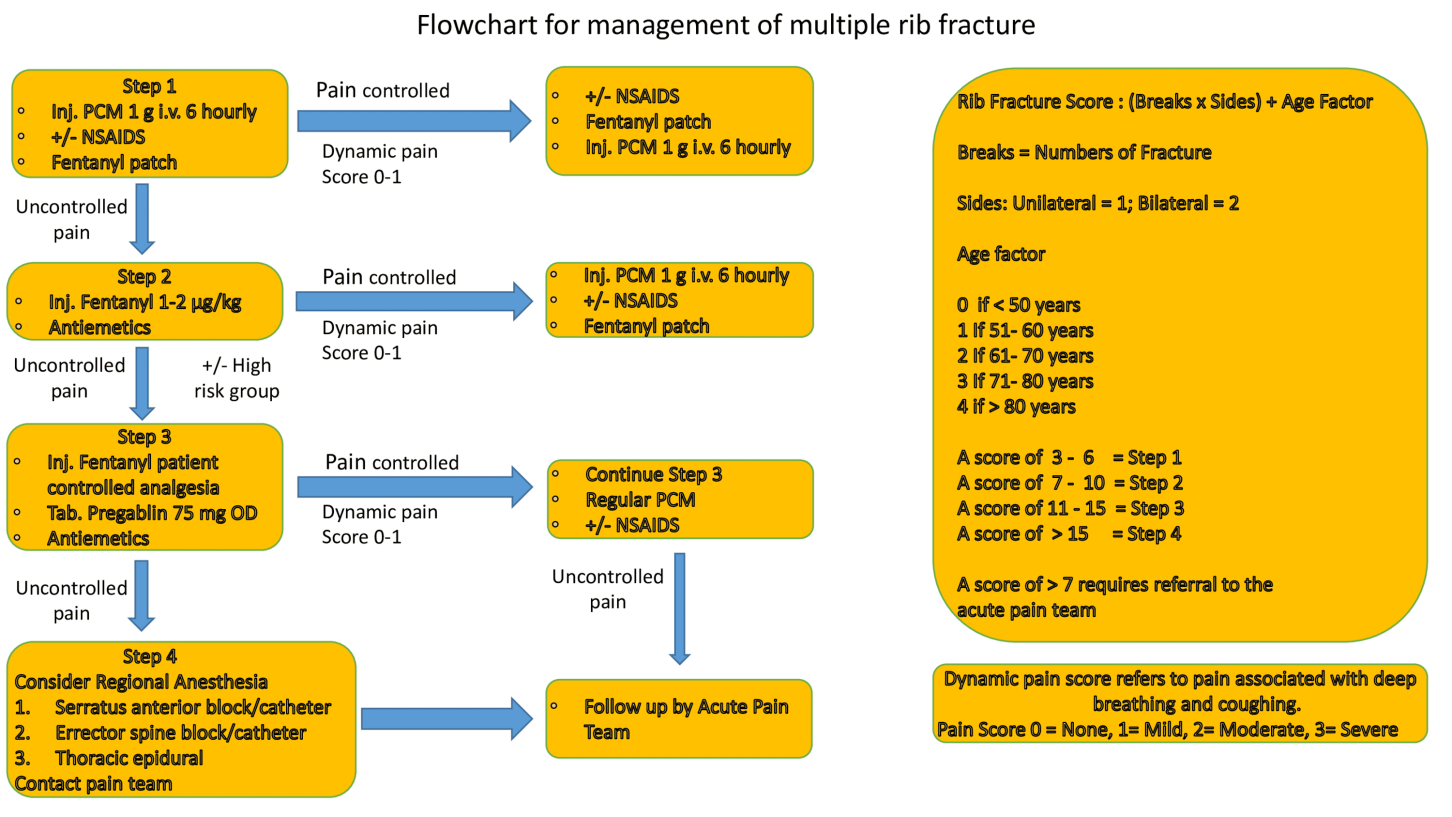
Flowchart for pain management in rib fracture Image credit: The image was created by the author Ganpat Prasad

Pain management was done with oral nonsteroidal anti-inflammatory drugs (NSAIDs), paracetamol 650 mg orally three times daily, or 1 g IV three times daily; opioids (morphine 10-20 mg twice daily, IV 0.1-0.2 mg/kg), and pregabalin 75 mg twice daily. Regional anesthesia is administered using an ultrasound-guided block of the erector spinae for posterior rib fracture. The initial loading dose of ropivacaine was set at 30 mL, followed by an infusion of 0.1% at 7 mL/hour with fentanyl at 1 µg/kg. The serratus anterior block is administered at the same dose as anterior rib fractures. For bilateral rib fractures, an epidural block with ropivacaine 0.2 in a dose of 10 mL is given, followed by an infusion of 5 mL/hour and fentanyl at 1 µg/kg (Figure 1).

The acquired data have been analyzed statistically employing descriptive statistics, including mean, median, and mode. The quantitative data were examined using the Student's t-test, while the qualitative data were evaluated using the chi-square test. The magnitude of the association has been demonstrated by the adjusted odds ratio (AOR) with a confidence interval (CI) of 95%. p values below 0.05 have been recognized as significant. The Kruskal-Wallis H test has been employed to compare the time taken to get to the hospital.

## Results

Of all trauma admissions, 1,576 patients (27.4%) had thoracic injuries. A male-to-female ratio of 3.9:1 has been observed among the patients, with 1,260 (79.9%) being male patients and 316 (20.1%) being female patients. The patients' ages ranged from 2 to 76 years old, with an average age of 41.62 years. The age range of 20-48 years comprised the majority of patients, 1,372 (87.1%). In 1,377 patients (87.4%), blunt trauma had been the most prevalent mechanism of injury; penetrating chest trauma had been reported by the remaining 199 patients (12.6%). In 819 patients (51.9%), traffic accidents constituted the prevalent trauma type, followed by falls from height (252; 15.9%), assault (189; 11.9%), low-energy falls (157; 10%), sports injuries (31; 2%), and others (126; 8%). Only 94 patients (6%) were admitted within the "golden hour," while the majority were reached up to 24 hours after the injury. Of the 1,576 patients, 1,308 (83%) were hemodynamically stable, while 268 (17%) were hemodynamically unstable. Isolated thoracic injuries were present in 724 patients (46%), while 852 (54%) were diagnosed with polytrauma. Eighteen percent of patients (283) reported subcutaneous emphysema at the time of presentation, and 33% (520) of patients had a positive chest X-ray. Single rib fractures were observed in 302 patients (19.2%), while multiple rib fractures occurred in 1,289 patients (81.8%). Unilateral thoracic injuries were observed in 1,127 (71.5%) and bilateral injuries in 449 patients (28.5%). Flail chest was found in 107 patients (6.8%). Pneumothorax was found in 614 patients (39%), hemothorax in 724 (45.9%), and pneumohemothorax in 238 patients (15.1%). The least frequent injuries include injuries to the esophagus in 9 (0.5%) and the heart in two patients (0.01%). Serious vascular injuries included the aorta in one patient (0.06%) and the subclavian vessels in five patients (0.03%). Extra thoracic injuries were found in 1,068 patients (67.8%). Overall, the mean injury severity score (ISS) has been 16.11 ± 6.54 with a median of 12. For blunt trauma, the mean ISS was 14.76 ± 8.1, while for penetrating trauma, it was 15.3 ± 4.8, and for polytrauma patients, it was 17.8 ± 6.1. International Classification of Diseases (ICD) insertion had been the most frequently performed surgical procedure in 1,338 (84%), unilateral in 1,057 (79%), and bilateral in 281 (21%). Tracheostomy was performed in 168 patients (10.6%) for prolonged ventilation. Respiratory support was provided to 890 patients (56.4%). Of these 890 patients, 739 (83%) only required oxygen by mask. Sixteen patients (1.7%) required noninvasive ventilation, while 135 patients (15.1%) were intubated and ventilated. Resuscitative thoracotomy was performed in two patients (0.12%) who had shock and pulseless electrical activity after penetrating injury. Thoracotomy was performed in 74 patients (4.7%). Video-assisted thoracoscopic surgery for empyema was performed in only three patients (0.01%). A multimodal strategy has been chosen to treat pain. NSAIDs and systemic opioids were administered to 1,136 patients (72%), while 430 (28%) received regional anesthesia. Of these 430 patients, 217 (50.4%) received a serratus anterior block, 139 (32.3%) received an erector spinae block, and 74 (17.2%) received epidural anesthesia. Of the 1,576 patients with thoracic injuries, 992 (63%) were treated without complications, whereas approximately 37% (583) suffered complications during hospitalization (Table 2).

**Table 2:** Thoracic injury sequelae MODS: multiple organ dysfunction syndrome

Complications	N (%)
Ventilator-associated pneumonia	62 (3.94)
Sepsis, MODS	41 (2.61)
Wound sepsis	30 (1.92)
Delayed pneumothorax	26 (1.67)
Retained hemothorax	17 (1.09)
Empyema	16 (1.02)
Acute renal failure	9 (0.6)
Acute pulmonary embolism	4 (0.29)
Pressure ulcer	7 (0.45)
Bronchopleural fistula	6 (0.39)

The average ICU stay was 26 days, ranging from 1 to 43 days. Forty-six patients (2.91%) had to be readmitted to the ICU. The median hospital stay was 18 days (1-64). The overall mortality rate was 119 (7.5%) (Table 3).

**Table 3:** Causes of death DIC: disseminated intravascular coagulation

Causes of death	Number (percentage of patients)
Irreversible hemorrhagic shock	55 (46.56)
Sepsis	32 (27.12)
Associated injuries including head injury	23 (19.81)
Respiratory failure	5 (3.87)
DIC	3 (2.61)
Pulmonary embolism	1 (0.8)

Mortality had been notably associated with increasing age, evidenced by 69 patients (57%) in the geriatric (age >50 years) group. The mortality rate among patients after surgery had been 35% (41 patients). The number of deaths within six hours of admission was 19 (15.9%), within 24 hours was 31 (26%), within 72 hours was 55 (46.2%), and after 72 hours was 14 (11.7%). The immediate cause of death was severe hemorrhagic shock, while type 1 respiratory failure and sepsis led to death in patients after 72 hours. Results of our research indicate the relationship among age, admission timing, penetrating injuries, bilateral chest injuries, concurrent extrathoracic injuries, and the necessity for mechanical ventilation (Table 4).

**Table 4:** Multivariate logistic regression analyses for factors associated with mortality CI: confidence interval; COR: crude odds ratio; AOR: adjusted odds ratio

Variable	N (%)	Odds ratio (95% CI)		p value
		COR (95% CI)	AOR (95% CI)	
Age				
<18	79 (5.0)	1	1	<0.001
19-35	491 (31.1)	1.19 (0.34-3.51)	1.63 (0.72-4.78)	
36-50	882 (55.9)	3.79 (2.46-5.91)	3.16 (0.89-7.31)	
>50	124 (7.8)	4.43 (3.57-9.35)	9.58 (4.12-26.19)	
Time between injury and admission				
<6 hours	94 (5.9)	1	1	<0.01
6-24 hours	346 (21.9)	2.21 (1.10-7.27)	2.22 (0.65-9.12)	
24-72 hours	598 (37.9)	3.15 (1.89-4.16)	3.81 (1.62-5.56)	
>72 hours	535 (33.9)	4.71 (0.98-11.74)	4.91 (0.71-8.24)	
Severity of injury				
Unilateral	1,127 (71.5)	1	1	<0.01
Bilateral	449 (28.4)	4.33 (2.71-8.97)	4.19 (1.57-16.29)	
Mechanism of injury				
Blunt	1,377 (87.3)	1	1	<0.001
Penetrating	199 (12.6)	1.69 (1.35-3.17)	3.96 (1.87-7.26)	
Mechanical ventilation needs				
Yes	119 (7.5)	12.41 (5.76-29.13)	11.03 (2.09-24.12)	<0.01
No	1,457 (92.4)	1	1	
Associated extrathoracic injury				
Yes	1,068 (67.8)	2.78 (1.91-5.31)	3.51 (1.62-8.71)	<0.01
No	508 (32.2)	1	1	

## Discussion

Current research aims to assess the impact of thoracic injuries and the resulting mortality and morbidity results. The prevalence of thoracic injuries in our research has been 27.4%, similar to reports from Galukande et al. [10] and Saidi et al. [11]. The research study indicated that most injured patients were males within the productive age group, with a male-to-female ratio of 3.9:1, consistent with conclusions from other researchers [12,13]. In accordance with previous research, we determined that road traffic accounted for the predominant proportion of thoracic injuries in our study [14-16]. Road traffic injuries continue to be among the primary causes of trauma within developing countries like India, mainly due to driving under the influence of recreational drugs, poor road structure, increasing motorization, and noncompliance with traffic laws; hence, there is a need for road traffic injury prevention measures [17]. In our study, most patients presented to a medical facility within a 24- to 72-hour period. Jegoda et al. indicated that the mean duration from occurrence to admission was 11 hours [18].

Our study demonstrated that individuals who arrived later exhibited higher mortality rates than those admitted earlier. The lack of a systematic ambulance service with first responders, as well as insufficient prehospital care, may account for delayed arrival along with increased fatality rates. In our research, the frequency of patients with blunt thoracic injuries was 87.4%, surpassing results from studies conducted in Uganda, Nigeria, the United Arab Emirates, and Tanzania [6,19,20]. Although penetrating thoracic injuries are less common than blunt trauma, they are more lethal. The risk of mortality was four times higher for penetrating thoracic injuries than for blunt injuries (AOR, 3.96; 95% CI, 1.87-7.26, p < 0.001). Regarding the type of thoracic injury, the majority of patients had hemothorax (724, or 45.9%), followed by pneumothorax (614, or 38.9%) and pneumohemothorax (238, or 15.1%). This result contrasts research by Adem et al., who found hemopneumothorax to be the prevalent type of thoracic injury (116, or 27.7%) in patients [21]. Our research demonstrated that 1,068 patients (67.8%) had associated injuries outside the chest. The most common associated injury was head injury (378, or 24%), followed by abdominal visceral injuries (305, or 19.4%). Shorr et al. illustrated associated injuries in more than 75% of patients with chest trauma [22]. Kulshrestha et al. reported a frequency of 1.5%-6% of cardiac injuries among chest trauma patients within a single department [23]. In our research, cardiac injury had been identified in about two patients (0.01%). Massaga et al. also reported similar results in a small number of patients with life-threatening conditions [5]. The majority of our patients had nonoperative treatment, aligning with results from earlier research [24,25]. Tube thoracostomy constituted the primary treatment modality for the majority of patients. Khursheed et al. reported a thoracotomy in 3.75% of patients and a tube thoracostomy in 65% [7]. In our study, thoracotomy has been conducted in 74 patients (4.7%), consistent with Richardson, which stated that less than 5% of patients with blunt chest trauma need thoracotomy [26]. The average length of ICD treatment had been 5.78 ± 3.21 days, with a median of five days (range, 1-27 days). LoCurto et al. reported a mean duration of thoracic drainage of 4.5 days [27]. During our research, the median duration of ICU admission had been 12.6 days, whereas the median hospital stay had been 18.09 days, contrasting with the research results of Veysi et al. [28]. Mechanical ventilation had to be performed for 135 patients (8.5%). Those who required mechanical ventilation had an 11-fold increased risk of death in regard to those who did not (AOR, 11.03; 95% CI, 2.09-24.12; p < 0.01). Numerous reviews indicated that hemorrhagic shock had been the leading reason for mortality among trauma patients [27]. We observed a mortality rate of 7.5% (119 patients) in the present research, which is identical to that reported by Mwesigwa et al. [6]. Battle et al. identified three primary risk factors for mortality in their systematic review and meta-analysis: age beyond 65 years, three or more rib fractures, and the existence of a preexisting disease [29]. Multiple research studies have highlighted that the elderly population experiences greater morbidity and mortality than younger age cohorts [5,28]. Among the most significant mortality predictors were age, bilateral chest injury, need for mechanical ventilation, and associated injuries, results that are almost consistent with those of other researchers. If a patient was older than 50, their chance of death increased by nine times (AOR, 9.58; 95% CI, 4.12-26.19, p < 0.001). Management improvement techniques that focus on these mortality factors are consequently required to improve the final results of thoracic injuries. The limitations of the study include that it was a single-center retrospective observation study with the presence of varying bias due to the study design.

## Conclusions

Thoracic injuries occur in a significant number of young patients of productive age. Road traffic injury accounted for the majority of thoracic trauma. Timely intervention, together with appropriate pain management, is required for improved outcomes. Associated injuries increase the risk of complications. It has been demonstrated that age, bilateral thoracic injuries, late admission lasting more than 24 hours, and the requirement for mechanical ventilation are indicators of risk for fatality in thoracic injuries.
